# Evaluation of Interactions of Added Soybean Peroxidase with Other Nutrients Present in Fish Feeds Using an In Vitro Digestive Simulation

**DOI:** 10.3390/ani13193046

**Published:** 2023-09-28

**Authors:** Wesclen Vilar Nogueira, María Jesús Aznar-García, Francisca P. Martínez-Antequera, Antonia M. Barros de Las Heras, Marcelo Borges Tesser, Jaqueline Garda-Buffon, Francisco Javier Moyano

**Affiliations:** 1 Escola de Química e Alimentos, Universidade Federal do Rio Grande, Rio Grande 96203-900, RS, Brazil; jaquelinebuffon@furg.br; 2 Departamento de Biología y Geología, Universidad de Almería, 04120 Almería, Spain; mag969@ual.es (M.J.A.-G.); fma996@inlumine.ual.es (F.P.M.-A.); abarros@ual.es (A.M.B.d.L.H.); 3 Instituto de Oceanografia, Universidade Federal do Rio Grande, Rio Grande 96203-900, RS, Brazil; mbtesser@gmail.com

**Keywords:** aflatoxin B_1_, biotechnology, oxidoreductase, pisciculture, mycotoxin, mitigation

## Abstract

**Simple Summary:**

Peroxidase (PO) is effective at mitigating or eliminating mycotoxins in fish feeds. However, the literature does not describe how the free radicals produced during oxidation by PO can interact with other nutrients and active compounds present in such feeds, affecting their bioavailability and modifying both the metabolism of the species and the quality of the final product. The objective of this study was to evaluate the impact of using PO as a treatment against mycotoxins on the nutritional quality of the fatty acids and polyphenols present in a fish feed. The results demonstrate that applying PO may have unwanted side effects on long-chain polyunsaturated fatty acids and the digestive bioaccessibility of polyphenols.

**Abstract:**

Peroxidase (PO) has been applied in different areas of industrial biotechnology, including the control of contaminants like aflatoxin B_1_ in fish feeds. However, its potential negative interactions with the macro and micro components of feeds have not been evaluated. The aim of this study was to evaluate the impact of PO’s addition to a feed on compounds like fatty acids and polyphenols using an in vitro simulation of the digestive tract of the tilapia. The influence on fatty acids was determined by changes in the peroxide index, with the feed including PO presenting values four times higher than those of the control feed. On the other hand, the in vitro digestive simulation also evidenced an effect of PO on the bioaccessibility of polyphenols significantly influenced by the total digestion time and temperature. The bioaccessibility of polyphenol ranged from 2.09 to 16.23 μmol of the total Trolox equivalent antioxidant capacity for the combinations evaluated in the study. The greatest bioaccessibility was observed at the central point under the following conditions of digestive hydrolysis: pH of 7, 30 °C, 4.5 h of digestive hydrolysis and an absence of PO.

## 1. Introduction

Fish farming has shown remarkable development, representing 17% of all animal protein produced worldwide [1]. This development is directly related to consumers’ search for healthy foods [2] and also to the high degree of mechanization and technologies employed within this sector, particularly innovations related to the development of artificial feeds able the cover the nutritional needs of fish in the various stages of their production cycle [3]. A great part of the ingredients used in such feeds are of plant origin, and in many parts of the world, they are highly susceptible to mycotoxin contamination [4,5]. Fish are highly sensitive to the effects of mycotoxins, and even if exposure is chronic and does not cause mortality, regular intake causes negative effects (e.g., liver disorder, low weight gain and low feed conversion) which affect productivity directly [6,7]. This requires not only extensive surveys to control their occurrence [8,9,10,11,12,13] but also developing strategies for mitigation of their negative effects on fish metabolism and to prevent contamination of fish products that will be consumed by humans [14].

Peroxidase (PO) comprises a group of nonspecific and specific enzymes (e.g., NADH peroxidase, glutathione peroxidase and iodine peroxidase) [15]. PO can be found in a wide variety of organisms (e.g., plants, microorganisms and animals) under different isoforms and are predominantly heme proteins containing iron (III) protoporphyrin IX as a prosthetic group [16]. These enzymes are considered multifunctional due to their ability to catalyze oxidation or polymerization reactions on a variety of substrates in the presence of hydrogen peroxide (H_2_O_2_) [15,17]. This feature allows PO to be applied in different areas of industrial biotechnology [18,19,20]. Different studies report the application of PO for humic acid inhibition [21], development of molecular markers [22], bleaching in the paper industry [20] and mitigation of contaminants (e.g., mycotoxins) in food and feed matrices [12,23,24,25,26].

Among enzymes, commercial peroxidase (PO) has shown that it is effective at mitigating mycotoxins in different feed matrices [27,28]. Other studies have shown that PO obtained from plant extracts (e.g., soy and rice bran) is effective at mitigating mycotoxins [23,29,30], and depending on the materials to which it is applied, it may reach better results than commercial enzymes [24]. However, there is a lack of studies describing how application of PO to animal diets to inactivate fungal contaminants can interact with other nutrients and active compounds, affecting their potential bioavailability and influencing both the metabolism of the species and the quality of the final product [31].

Among the components present in fish diets that can be negatively affected by PO oxidation, there are some important molecules, such as long chain polyunsaturated fatty acids (LC-PUFA) and polyphenols [31,32,33,34]. LC-PUFA are considered key nutrients for a great number of fish species due to their roles in maintaining homeostasis, as well as the functionality of the reproductive and immune systems [35,36]. Nevertheless, due to the presence of several unsaturated bonds in their carbon chains [37], LC-PUFA are highly susceptible to peroxidation that can compromise the integrity of such bonds and hence their functionality [38].

Polyphenols are secondary metabolites of plants and are generally involved in defense against ultraviolet radiation or aggression. Increasing attention is currently being paid to the protective roles of polyphenols obtained from different sources in health and oxidative status in fish, since in addition to influencing the immune system, they present antioxidant, anti-inflammatory, immunomodulatory and hepatoprotective effects [39,40,41,42], aside from influencing growth and feed conversion efficiency [43] as well as the composition of the intestinal microbiota [44].

Therefore, on one hand, the inclusion of PO in fish feeds may be beneficial for minimizing the negative effects of aflatoxins, and on the other, due to its high reactivity, it may negatively interact with the nutritional quality of the lipid fraction as well as polyphenols included in such feeds, thus reducing their beneficial effects. Nevertheless, since it may be difficult to assess such effects in vivo, alternative approaches can be used to obtain a preliminary estimation of the nature and extension of such interactions. In this sense, in vitro methodologies simulating the physiological conditions present in the digestive tract of fish, that have been widely applied to evaluate bioaccessibility of nutrients present in fish feeds [45] can be highly valuable. This evaluation is particularly important in the case of intensive systems of production, in which all the nutrients required for the development and growth of the animals are provided exclusively by the feed [46].

Considering the above-described points, the objective of this study was to evaluate the impact of using PO as a treatment against mycotoxins on the stability and potential bioavailability of fatty acids and polyphenols present in a fish feed. This study was based on the use of an in vitro model simulating the digestive tract of tilapia (*Oreochromis niloticus*), with the selection of this species justified by its relevance in freshwater aquaculture worldwide as well as the great use of plant ingredients susceptible to mycotoxin contamination in its feeds.

## 2. Materials and Methods

The present study was developed through two different experiments aimed to (1) evaluate the potential oxidative effect of PO on the lipid fraction of a feed and (2) evaluate how the interactions of PO with the polyphenols included in a feed may affect their digestive bioaccessibility under different digestion conditions.

### 2.1. Supplies, Chemicals and Reagents

Soybean meal used for PO extraction was provided by a company specialized in soybean processing located in the municipality of Rio Grande (Rio Grande do Sul, Brazil). Grape pomace was provided by the Bodega artisanal located in the municipality of Fondón (Almeria, Spain). Sodium phosphate mono and dibasic were purchased from PanReac AppliChem (Darmstadt, Germany) and Merck (Darmstadt, Germany), respectively. Guaiacol and hydrogen peroxide were purchased from Sigma (Steinheim, Germany). Tannic acid, 2,2-diphenyl-1-picrylhydrazyl (DPPH), (±)-6-Hydroxy-2,5,7,8-tetramethylchromane-2-carboxylic (Trolox) and carboxymethylcellulose (CMC) were purchased from Sigma (Saint Louis, MO, USA). Butylhydroxytoluene (BHT) was obtained from Merck (Darmstadt, Germany). Potassium chloride was obtained from PanReac AppliChem (Barcelona, Spain). Chloroform was purchased from Fisher Scientific (Mallinckrodt, Hampton, NH, USA). Methanol and potato starch were purchased from PanReac AppliChem (Darmstadt, Germany). Sodium sulfate and bovine albumin were purchased from Sigma (Steinheim, Germany).

### 2.2. Obtaining and Determining the PO Activity

The soybean meal PO extraction conditions were established according to the method described by Feltrin et al. [23], using phosphate buffer with a pH of 4.7 as the extracting solvent. The supernatant obtained after bran homogenization with the buffer and centrifugation (3220× *g*, 10 min, 4 °C) was filtered for evaluation of the enzymatic activity and protein content. The enzymatic activity was verified in a spectrophotometer at 470 nm through the oxidation of guaiacol substrate to tetraguaiacol. A unit (U) of PO activity was defined as the amount of enzyme that catalyzed the oxidation of 1 μmol of guaiacol per minute under the test conditions. The specific activity of the PO was determined in relation to the amount of soluble protein (mg mL^−1^) determined by the method of Lowry [47], using bovine serum albumin as a standard.

### 2.3. Experiment 1: Evaluation of the Potential Oxidative Effect of PO on the Lipid Fraction of a Feed

#### 2.3.1. Feed Preparation

A model simplified diet was designed to carry on the experiments, being prepared with a few pure ingredients to avoid possible interference in the colorimetric measurements required for the assays. Four different formulations were prepared including or not including PO as well as BHT, an antioxidant compound routinely used in aquafeeds to assess the ability of this compound to counteract the oxidative effect produced by PO. The amount of BHT used was in accordance with that recommended by Lundebye et al. [48]. The compositions of the diets are detailed in Table 1. Diets including PO were processed following the optimal conditions determined by Nogueira et al. [12] when using the enzyme as a pretreatment of the feeds to mitigate aflatoxin B_1_ (0.035 U g^−1^, 70% moisture, 32 °C for 2 h). For all formulations, solid ingredients were mixed from the lowest to the highest ratio. Then, liquid ingredients were added. The ingredients were manually homogenized to form a uniform mass, followed by incubation for 2 h at 32 °C. The masses resulting from all treatments were pelleted by a bench grinder. The pellets were dried in an oven at 60 °C for 8 h, packaged, identified and kept at −4 °C until used for the experiments.

For the diets containing BHT, this was previously diluted in the oil fraction and then mixed with the rest of the ingredients. Once prepared and dried, samples (5 g) of the different diets were used to extract the lipid fraction and to measure the degree of oxidation.

#### 2.3.2. Extraction of the Lipid Fraction and Determination of the Peroxide Value

The feeds were subjected to extraction of the lipid fraction through the method of Folch et al. [49] using a mixture of chloroform and methanol as the extracting solvent (3:2, v v^−1^). The determination of the peroxide index followed the methodology established by Instituto Adolfo Lutz [50] with some modifications. For this, 250 mg of lipid was added in an Erlenmeyer flask (50 mL) with 7.5 mL of acetic acid:chloroform (3:2, v v^−1^) and homogenized until dissolution. Then, 125 μL of saturated potassium iodide solution was added, and the reaction was kept in the dark for 1 min. Then, 250 μL of starch solution (1%) was added and titrated with sodium thiosulphate (0.1 or 0.01 N).

### 2.4. Experiment 2: Assessing How Interaction of Dietary Polyphenols with Ps May Affect Their Bioaccessibility under a Variety of Digestion Conditions

This experiment was developed in two parts: (1) A preliminary quantitative evaluation of the negative interaction of PO with the antioxidant capacity of the polyphenols present in a natural source (grape pomace) and (2) an evaluation of different factors that may significantly affect the digestive bioavailability of polyphenols after preincubation of PO together with a model polyphenol (tannic acid) within a feed matrix.

#### 2.4.1. Antioxidant Capacity of Grape Pomace Extract

Grape pomace (GP) was used as a natural source of polyphenols. The GP was dried in an oven for 72 h at 60 °C and subsequently finely grounded in a knife mill (Silver Crest, HG08263, Bochum, Germany) until obtaining a particle size ≤0.5 mm. Characterization of the phenolic compounds was carried out according to Veskoukis et al. [51] with modifications. For this, we used an aqueous extract prepared by mixing 0.5 g GP with 4.5 mL of distilled water. The extraction was carried out in an oven (60 °C for 180 min) with vortex agitation every 30 min in the first hour and every 60 min in the following 2 h. The extract was then centrifuged at 3000× *g* for 15 min to obtain the supernatant containing the bioactive compounds. The Trolox equivalent antioxidant capacity (TEAC) was determined by following the DPPH and ABTS method described by Brand-Williams et al. [52] and Re et al. [53], respectively.

#### 2.4.2. Inhibition of the TEAC Present in Grape Pomace by PO

This trial was carried out to evaluate the inhibitory effect of soybean meal PO on the TEAC present in GP extract. For this, a dose/response assay was designed by incubating a fixed amount of GP providing 171.7 μmol of TEAC (mL^−1^), with different amounts of a solution providing changing activities of PO: 0.01; 0.035; 0.06; 0.085 and 0.1 U mL^−1^. The assays were carried out using 2 mL Eppendorf tubes containing glass spheres to increase the interaction between the molecules. After adding the enzyme, both solutions were incubated at 32 °C for 2 h with rotary stirring (150 rpm) (NR50 E, Ovan, Barcelona, Spain). At the end of the process, the antioxidant capacity of each test was determined according to Section 2.4.1, and the data were compared with a blank prepared without PO.

#### 2.4.3. Changes in the Potential Bioavailability of a Polyphenol Included in the Feed after Pretreatment with PO

The feed matrix used to develop the different in vitro digestion assays was prepared using the same ingredients and conditions described in Section 2.3.1. In addition, pure tannic acid was added as a model polyphenol, with the added amount (3%) being based on studies by Buyukcapar et al. [54]. One feed did not include PO, while the other was treated with the enzyme using the amounts and conditions described in Section 2.3.1.

##### Tilapia Digestive Enzyme Extracts

The enzymatic extract used in the tests was obtained from adult specimens of tilapia (*O. niloticus*). The fish (n = 60; total biomass of 2148.9 g; individual weights ranging from 17.8 to 69.61 g and average weight of 35.8 ± 11.29 g) were kept in 500 L circular tanks with a skimmer, aeration supplementation, temperature control (19–22 °C) and photoperiod set at 12:12 h (light:dark) for 2 weeks. The pH and dissolved oxygen levels ranged from 6.5 to 7.0 and from 6.0 to 7.3 mg L^−1^, respectively. The animals were fed twice a day (8:00 a.m. and 5 p.m.) until apparent satiation with commercial feed containing 35% crude protein. At the end of a 2 week period, the fish were sacrificed 4 h after receiving a meal by immersion in a buffered solution of benzocaine hydrochloride (500 ppm) and dissected to obtain the digestive tract. Tissues were mechanically homogenized with distilled water (1:10, w v^−1^), followed by centrifugation at 3220× *g* for 20 min at 4 °C, with the supernatant filtered and concentrated using a dialysis system with 10 kDa MWCO (Pellicon XL, Millipore^®^, Burlington, MA, USA) coupled to a peristaltic pump (Minipuls^®^ 3, Gilson, Lewis Center, OH, USA). Concentrated extract was then lyophilized and stored at −20 °C until being used in the assays. The total activity of alkaline proteases in the lyophilized extract (U g^−1^ of fish) was measured using casein as a substrate according to the method described by Kunitz [55] and modified by Walter [56]. For further details on the procedure and determination of enzymatic activities, see the work of Morales and Moyano [57].

##### In Vitro Assays

In vitro assays involving the stomach and intestinal stages were carried out using membrane bioreactors [57]. The device consisted of two chambers separated by a 3500 kDa MWCO semi-permeable membrane (ServaPor^®^, Heidelberg, Germany). In the upper part, water, feed, enzyme extract and phosphate buffer were added and maintained under continuous agitation with the aid of a magnetic stirrer (300 rpm). The hydrolysis products were permeated through the membrane into the lower chamber containing a phosphate buffer and recovered for determination of the TEAC and ABTS according to Section 2.4.1. All assays maintained the same volume conditions in the upper and lower parts of the chamber, being 5 and 15 mL, respectively. The assays were carried out under physiologically based conditions, using enzyme-to-substrate ratios that considered the total amount of alkaline protease measured in a fish of 25 g (1.53 U g^−1^), as well the average consumption of feed by a fish of such a size if received in a single meal (1% of body mass) containing 45% crude protein.

##### Experimental Design

Evaluation of different factors that can significantly affect the digestive bioavailability of polyphenols after preincubation of PO, and a model polyphenol (tannic acid) within a food matrix described in Section 2.4 was developed using an in vitro simulation of the tilapia digestive tract and carried out using a Plackett–Burman (PB) factorial design to determine which factors could be significant in this interaction.

This study considered two fixed factors (stomach pH and residence time) and four variable factors (inclusion or not of PO in the feed matrix, intestinal pH, reaction temperature and total digestion time) (Table 2). This resulted in a total of 18 runs (one block and one replicate each. The response variable measured was the estimated potential bioavailability of the polyphenol (tannic acid) after digestion, expressed in μmol of the total TEAC. The values of the stomach pH (5.0) and residence time (1 h) were based on those described by Uscanga et al. [57] for tilapia juveniles. Physiological ranges of the intestinal pH (6.0–8.0), temperature (25–35 °C) and total digestion time (3–6 h) were established according to published data for tilapia [58,59,60,61].

### 2.5. Statistical Analysis

The assumptions of homoscedasticity and normality were tested for the results obtained. Statistical analysis of the effect of PO on lipid oxidation (n = 3) was carried out by one-way ANOVA followed by Tukey’s test at *p* ≤ 0.05. Both the PB factorial design and analysis of results were carried out with Minitab^®^ 16 software (Minitab Inc., State College, PA, USA).

## 3. Results

### 3.1. The Effect of PO on Fatty Acids Present in the Feed

The oxidative effect of PO on the lipid fraction of the feed was evidenced by a significantly higher (*p* ≤ 0.05) peroxide index (Table 3). Nevertheless, when present in the mixture, BHT clearly counteracted the negative effect of PO on lipid oxidation.

### 3.2. The Inhibitory Effect of PO on the Bioactive Compounds of the Extract of Grape Pomace

**Figure 1:**
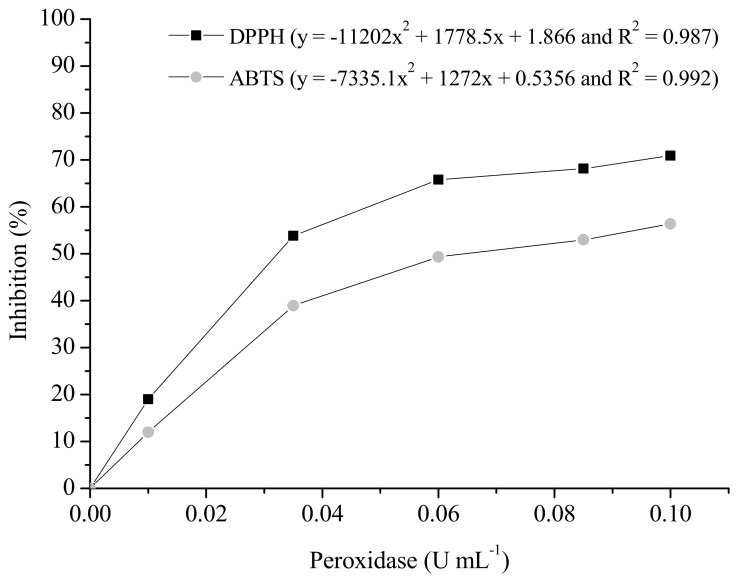
Inhibitory effect of different PO concentrations on bioactive compounds in grape extract.

The results of the preliminary assays of inhibition by PO of the bioactive compounds initially present in GP (171.7 μmol of TEAC mL^−1^), determined by DPPH and ABTS, are shown in Figure 1. A similar quadratic response was obtained in both cases, although the values measured using ABTS were significantly lower. Maximum inhibition of the oxidative response was obtained in both cases at levels of PO between 0.08 and 0.1 U mL^−1^.

### 3.3. PB Design for Evaluating Polyphenol Inhibition with PO

The values of the TEAC provided by tannic acid resulting from the different combinations of factors ranged from 2.09 to 16.23 μmol (Table 4). The greatest bioaccessibility was observed at the combination generated with central values, with a pH of 7, temperature of 30 °C, 4.5 h of digestive hydrolysis and the absence of PO.

The results of the ANOVA with the regression coefficient (RC), standard error (SE) and *t* and *p* values obtained in the PB design for polyphenol bioaccessibility are presented in Table 5. The R^2^ values suggest that only 4.24% of the variations in the bioaccessibility of polyphenol (*Y*) could not be explained by the selected independent variables (*X*_1_–*X*_4_). Temperature (*X*_2_) had the most significant negative effect on polyphenol bioaccessibility (*X*_2_ = −1.928, *p* < 0.001). The other variables showed a positive effect, but pH (*X*_1_) did not show statistical significance in the bioaccessibility of polyphenol (*p* > 0.05). A negative value for the temperature (*X*_2_) means that the change from a level of −1 (25 °C) to +1 (35 °C) reduced the bioaccessibility of polyphenol, whereas the positive effect observed in the digestion time (*X*_3_) means that the change in the variable from a level of −1 (3 h) to +1 (6 h) increased bioaccessibility. Furthermore, the higher the *t* value and the lower the *p* value, the more significant the regression coefficient and influence on the process. Therefore, the presence (+1) or absence (−1) of PO (*X*_4_) had a direct impact on the bioaccessibility of the polyphenol present in the diet.

## 4. Discussion

The results of Experiment 1 demonstrate that the presence of PO determined a significant peroxidation of the lipids included in the feed (Table 3) and also that this negative effect can be counteracted in the presence of a radical scavenger like BHT, a synthetic derivation of vitamin E widely applied in fish feeds due to its stability, low cost and high availability [62]. The mechanism by which BHT acts is directly related to the removal of free radicals from the environment, interfering in the propagation of the oxidation reaction responsible for the alteration of lipids [63]. Considering this, from a practical point of view, the treatment of plant ingredients with PO should be carried out sequentially prior to addition of the lipids (which should include BHT) in order to avoid interference between the required oxidation provided by PO and the protective effect of BHT on unsaturated fatty acids present in the lipid fraction.

Also, it must be noted that although there are no studies on using BHT as a protective agent against AFB_1_ in fish, its use is related to inhibition of the negative effects of this mycotoxin, such as bioavailability and inhibition of hepatocarcinogenesis in birds [64] and rodents [65], respectively. Although the protective properties of BHT are attributed to the induction of glutathione S-transferases and other enzymes involved in AFB_1_ metabolism, part of its protective effect may be related to its ability to inhibit hepatic epoxidation of AFB_1_ mediated by cytochrome P450 to exo-AFB_1_-8,9-epoxide [64]. Furthermore, BHT-induced hydropic degeneration is reversible, not causing long-term damage if animal species are exposed [66]. Therefore, BHT can neutralize the oxidative effect of PO within the fish feed production sector and contribute to reducing the damage caused by AFB_1_ to cultivated species.

The results in Experiment 2 demonstrate that preliminary treatment of feed ingredients with PO may negatively affect the antioxidant capacity of polyphenols, irrespective of if they were already present in such ingredients or specifically added as functional additives. In fact, due to their high reactivity with phenolics, peroxidases are used to remove these compounds in depuration of waste waters, since such enzyme treatment offers a high degree of specificity, operation under mild conditions and a high reaction velocity [67]. Also, interactions between peroxidase and phenolic compounds, resulting in modifications of their chemical structure and bioavailability, have been demonstrated in different plant ingredients [68,69].

In the present study, the negative interaction between PO and the phenolic compounds present in grape pomace was evidenced by a significant reduction in the TEAC (Figure 1). This can occur through dissociation of the hydroxyl groups (-OH) that are linked to the aromatic rings of the molecules, allowing the formation of radicals by hydrogen abstraction. In this oxidation process, intermediate radicals of simple polyphenolic molecules can originate complex and insoluble molecules in an aqueous medium [70], which would make their digestive hydrolysis impossible, thus preventing their quantification under the conditions used in this study.

On the other hand, the results obtained with the in vitro assays evidenced that the potential digestive bioavailability of dietary polyphenols may be significantly reduced if PO is used as an additive and also that the total length of the digestion process may positively influence such bioavailability, while a higher water temperature decreases it. The positive effect of a longer digestion time can be easily understood, since it correlates directly to a higher exposition of the feed matrix to the action of the digestive enzymes and hence to an increased opportunity for polyphenols to be released, even if a fraction of them has been irreversibly inactivated after their initial interaction with PO. In a recent study by Martínez-Antequera et al. [71], they developed in vitro assays to determine the bioaccessibility of wine polyphenols, simulating the digestion of two fish species with different feeding habits (omnivorous (*Sparus aurata*) and herbivorous (*Mugil cephalus*)). They demonstrated that a longer digestion time increased the bioavailability of different types of polyphenols and also that such a positive effect cannot be generalized to all the compounds present in wine bagasse. It follows that, in spite of the positive effect of a longer digestion time observed in the present study when using one type of polyphenol (tannic acid), a proper evaluation of the net effect of the digestion time on the potential bioavailability of a given polyphenol after interaction with PO should require specific assays. On the other hand, it can be deduced that rapid intestinal transit rates can significantly affect the bioaccessibility of bioactive compounds. Herbivorous fish typically have longer intestines but higher gastrointestinal transit rates than carnivorous or omnivorous species [72]. Although there is not much available information on gut evacuation rates for tilapia, the estimated time is 7 h for juveniles (30 g) kept at 28 °C and fed twice daily [58]. This may represent a limitation for maximizing digestive release and the absorption of phenolic compounds if the feed has been treated with PO to counteract the effect of aflatoxins.

The negative effect of an increasing temperature on the potential bioavailability of the polyphenol obtained in the model was more surprising. In the live fish, a higher temperature should have resulted in a general activation of metabolism, including increased activity of the digestive enzymes, which should have improved the release of tannic acid from the feed matrix. In contrast, the observed reduction in the total TEAC could be explained by considering that the formation of complexes between the highly reactive tannic acid and other components of the feed (protein and carbohydrates) could be enhanced at a higher temperature [73,74,75]. Again, the conditions routinely used for the culture of tilapia, with water temperatures frequently reaching 30–32 °C, may limit the digestive availability of dietary polyphenols if feeds are treated with PO.

## 5. Conclusions

Practical application of PO as pretreatment to eliminate aflatoxins of plant ingredients used in fish feeds may present undesirable side effects in some highly reactive dietary compounds like LC-PUFA or polyphenols. The negative effects on the former can be avoided by using sequential processing, with the initial treatment of plant ingredients with PO followed by addition of the lipid fraction including BHT as a protective agent. Negative interactions with polyphenols taking place during feed preparation has an impact on further digestive bioavailability of such compounds, with the latter negatively affected by short gut transit times and high water temperatures, conditions that may be frequent in fish species reared in warm regions (i.e., tilapia). These results must be considered in the optimization of the treatment with PO as well as knowing the doses of polyphenol sources provided with the feed.

## Figures and Tables

**Table 1:** Ingredients used to formulate experimental diets.

Ingredients	Control	PO	BHT	PO + BHT
Albumin (%)	45	45	45	45
Sunflower oil (%)	9	9	9	9
Fish oil (%)	9	9	9	9
Potato starch (%)	10	10	10	10
CMC (%)	15	15	15	15
PO (U g^−1^)	*	0.035	*	0.035
BHT (mg kg^−1^)	*	*	100	100

* = not included.

**Table 2:** Coded and actual values used in PB planning.

Factor	Coded Factor	Level	
		−1	+1
pH	*X*_1_	6	8
Temperature (°C)	*X*_2_	25	35
Time (h)	*X*_3_	3	6
Peroxidase (U g^−1^)	*X*_4_	Presence	Absence

**Table 3:** Effect of PO on the oxidation of fatty acids present in the feed.

Feed	Peroxide Index (meq kg^−1^)
Control	13.33 ± 0.05 ^a^
PO	50.67 ± 0.15 ^b^
BHT	10.67 ± 0.05 ^a^
PO + BHT	13.33 ± 0.11 ^a^

Results presented as mean ± coefficient of variation of triplicate assays. Equal letters in the same column demonstrate the absence of significant differences between treatments at the assessed significance level (*p* ≤ 0.05).

**Table 4:** PB design with coded and actual values (in parentheses) and responses (*Y*) for the study of polyphenol bioaccessibility in fish feed.

Assay	Encoded Values (Real Values)				Bioaccessibility of Polyphenol (*Y*) in μmol of Total TEAC
	*X*_1_ (*x*_1_)	*X*_2_ (*x*_2_)	*X*_3_ (*x*_3_)	*X*_4_ (*x*_4_)	
1	1 (8)	1 (35)	−1 (3)	1 (Absence)	6.51
2	1 (8)	−1 (25)	−1 (3)	−1 (Presence)	4.61
3	−1 (6)	−1 (25)	−1 (3)	−1 (Presence)	4.55
4	1 (8)	1 (35)	1 (6)	−1 (Presence)	4.53
5	1 (8)	−1 (25)	1 (6)	1 (Absence)	10.50
6	−1 (6)	1 (35)	1 (6)	−1 (Presence)	2.09
7	1 (8)	−1 (25)	1 (6)	1 (Absence)	10.98
8	−1 (6)	1 (35)	1 (6)	1 (Absence)	6.45
9	−1 (6)	1 (35)	−1 (3)	1 (Absence)	4.27
10	1 (8)	1 (35)	−1 (3)	−1 (Presence)	2.48
11	−1 (6)	−1 (25)	−1 (3)	1 (Absence)	10.14
12	−1 (6)	−1 (25)	1 (6)	−1 (Presence)	8.68
13	0 (7)	0 (30)	0 (4.5)	0 (Absence)	15.94
14	0 (7)	0 (30)	0 (4.5)	0 (Absence)	16.23
15	0 (7)	0 (30)	0 (4.5)	0 (Absence)	16.16
16	0 (7)	0 (30)	0 (4.5)	0 (Presence)	10.12
17	0 (7)	0 (30)	0 (4.5)	0 (Presence)	10.66
18	0 (7)	0 (30)	0 (4.5)	0 (Presence)	10.55

*X*_1_ = pH; *X*_2_ = temperature (°C); *X*_3_ = time (h) and *X*_4_ = peroxidase (U g^−1^).

**Table 5:** Regression coefficient (RC), standard error (SE) and *t* and *p* values obtained in the PB design for bioaccessibility of bioactive compounds.

Factor	RC	SE	*t*	*p*
*X*_1_	0.286	0.320	0.890	0.389
*X*_2_	−1.928	0.320	−6.030	<0.001
*X*_3_	0.890	0.320	2.780	0.017
*X*_4_	2.162	0.261	8.280	<0.001
R^2^	0.9576			
R^2^_ad_	0.9400			
R^2^_pr_	0.8952			

*X*_1_ = pH, *X*_2_ = temperature (°C), *X*_3_ = time (h), *X*_4_ = peroxidase (U g^−1^), R^2^ = determination coefficient, R^2^_ad_ = adjusted coefficient of determination and R^2^_pr_ = predicted coefficient of determination.

## Data Availability

The data presented in this study are available upon request from the corresponding author.
